# Remission of Diabetes Following Bariatric Surgery: Plasma Proteomic Profiles

**DOI:** 10.3390/jcm10173879

**Published:** 2021-08-28

**Authors:** María Insenser, Nuria Vilarrasa, Joan Vendrell, Héctor F. Escobar-Morreale

**Affiliations:** 1 Diabetes, Obesity and Human Reproduction Research Group, Instituto Ramón y Cajal de Investigación Sanitaria (IRYCIS), Hospital Universitario Ramón y Cajal, Universidad de Alcalá, E-28034 Madrid, Spain; mariarosa.insenser@salud.madrid.org; 2 Centro de Investigación Biomédica en Red Diabetes y Enfermedades Metabólicas Asociadas (CIBERDEM), E-28029 Madrid, Spain; nuriavilarrasa@yahoo.es (N.V.); jvortega2002@gmail.com (J.V.); 3 Department of Endocrinology & Nutrition, Hospital Universitari Bellvitge, Hospitalet de Llobregat, E-08907 Barcelona, Spain; 4 Department of Endocrinology & Nutrition, Institut d’Investigació Sanitaria Pere Virgili, Hospital Universitari de Tarragona Joan XXIII, E-43005 Tarragona, Spain

**Keywords:** 2D-DIGE, bariatric surgery, diabetes remission, obesity, proteomics, type 2 diabetes

## Abstract

Bariatric surgery restores glucose tolerance in many, but not all, severely obese subjects with type 2 diabetes (T2D). We aimed to evaluate the plasma protein profiles associated with the T2D remission after obesity surgery. We recruited seventeen women with severe obesity submitted to bariatric procedures, including six non-diabetic patients and eleven patients with T2D. After surgery, diabetes remitted in 7 of the 11 patients with T2D. Plasma protein profiles at baseline and 6 months after bariatric surgery were analyzed by two-dimensional differential gel electrophoresis (2D-DIGE) and matrix-assisted laser desorption/ionization-time-of-flight/time-of-flight coupled to mass spectrometry (MALDI-TOF/TOF MS). Remission of T2D following bariatric procedures was associated with changes in alpha-1-antichymotrypsin (SERPINA 3, *p* < 0.05), alpha-2-macroglobulin (A2M, *p* < 0.005), ceruloplasmin (CP, *p* < 0.05), fibrinogen beta chain (FBG, *p* < 0.05), fibrinogen gamma chain (FGG, *p* < 0.05), gelsolin (GSN, *p* < 0.05), prothrombin (F2, *p* < 0.05), and serum amyloid p-component (APCS, *p* < 0.05). The resolution of diabetes after bariatric surgery is associated with specific changes in the plasma proteomic profiles of proteins involved in acute-phase response, fibrinolysis, platelet degranulation, and blood coagulation, providing a pathophysiological basis for the study of their potential use as biomarkers of the surgical remission of T2D in a larger series of severely obese patients.

## 1. Introduction

Type 2 diabetes (T2D) is common in patients with severe obesity in whom a marked weight loss frequently restores glucose tolerance [1]. Randomized controlled trials with postoperative follow-up ranging from 1 to 5 years documented sustained diabetes remission in 30–63% of patients submitted to bariatric surgery [2,3,4], with similar remission rates regardless of specific surgical procedures [5,6]. Better postoperative outcomes are associated with younger age, shorter duration of diabetes, lack of need of insulin administration, better glycemic control, and certain gut microbiota profiles [7,8,9]. Metabolic benefits may appear very soon after surgery, well before significant weight loss occurs [10], by mechanisms that, in addition to improvement in beta-cell function and normalization of hepatic glucose production [10,11,12], are not fully understood.

Even though several scores have been proposed to predict diabetes remission after metabolic surgery [13,14,15,16,17], none are actually effective. The present study focused on the identification of the early determinants of the resolution of T2D in severely obese patients after bariatric surgery by applying a non-targeted proteomic analysis to plasma proteins.

## 2. Materials and Methods

### 2.1. Subjects of Study

Seventeen severely obese women, including six metabolically healthy subjects and eleven patients with T2D, were included in the present study. The diagnosis of diabetes relied on the American Diabetes Association guidelines [18]. Remission of diabetes after surgery required glycated hemoglobin <6% and fasting plasma glucose <100 mg/dL in patients not taking antidiabetic drugs [19]. Surgical procedures and perioperative management followed international guidelines for obesity surgery [20,21,22]. Fourteen patients were submitted to Roux-en-Y gastric bypass and three were submitted to sleeve gastrectomy.

All individuals underwent a comprehensive clinical, anthropometric, and physical evaluation and a complete biochemical analysis, before and 6 months after surgery. Written informed consent was obtained from all the participants and the study was approved by the local ethics committees of the participating hospitals.

### 2.2. Sample Preparation and Two-Dimensional Differential in Gel Electrophoresis 2D-DIGE

Proteins were extracted from plasma and albumin, and IgG was removed with commercial removal kits (GE Healthcare, Buckinghamshire, U.K.). Protein labeling, two-dimensional differential in gel electrophoresis (2D-DIGE), image analysis, and protein identification were already described [23] and are detailed in the Supplementary Data Text, Supplementary Table S1 and the MIAPE document.

### 2.3. Enzyme-Linked Immunoassays and Immunenephelometry Assays

Six proteins identified in the non-targeted proteomic analysis were assayed by enzyme-linked immunoassays (ELISA): (serum amyloid P (APCS), alpha-1-antichymotrypsin (SERPINA3), kininogen (KNG), and inter alpha globulin inhibitor H4 (ITIH4)) or immunonephelometry: ceruloplasmin (CP) and alpha-1-antitrypsin (SERPINA1). The assays used for these measurements are described in detail in Supplementary Data Text.

### 2.4. Statistical Analysis

Data are expressed as means ± SD unless otherwise stated. After testing continuous variables for normality using the Kolmogorov–Smirnov test, we applied logarithmic transformation as needed to ensure the normality of skewed variables. Data from plasma samples were submitted to univariate one-way or repeated-measures general linear models (GLMs) as appropriate, which were followed by the post hoc least significant difference test for multiple comparisons to identify differences among the group of patients (PASW Statistics 18, SPSS, Inc., Chicago, IL, USA). Time of sampling (before surgery and after 6 months of follow-up) was introduced as the within-subjects effect, and the group of subjects (no diabetes before and after surgery, diabetes remission after surgery, and persistent diabetes after surgery) was used as the between-subjects effect. The interaction among the within- and between-subjects effects was analyzed. The level of significance was set at *p* < 0.05.

## 3. Results

### 3.1. Clinical Variables before and after Surgery

Six patients did not have diabetes before surgery (non-diabetic group). Eleven patients presented with diabetes before surgery; glucose tolerance returned to normal after 6 months in seven of them (diabetes remission group), whereas diabetes persisted in the other four (persistent diabetes group). Table 1 summarizes their clinical and biochemical characteristics, respectively.

We found no statistically significant differences between groups in age, BMI, waist circumference, body fat percentage, fasting insulin, triglycerides total-cholesterol, or LDL-cholesterol concentrations before surgery. Fasting plasma glucose and glycated hemoglobin were lower in the control group compared with both groups of patients with diabetes. HOMA-IR was also lower in the non-diabetic group compared with both groups of patients with diabetes, even though this tendency did not reach statistical significance between the non-diabetic and persistent diabetes groups. HDL-cholesterol concentrations were reduced in the diabetes remission group compared with non-diabetic patients. The counts of patients submitted to gastric sleeve or gastric bypass procedures were one and six in the non-diabetic group, two and seven in the diabetes remission group, and zero and four in the persistent diabetes group, respectively.

We found no statistically significant differences among the groups in the decrease in BMI observed six months after surgery or in the percentage of excess weight loss (Table 1a). All metabolic markers decreased, except for HDL-cholesterol concentrations, which did not change (Table 1b). We observed an interaction between the group of patients and bariatric surgery in fasting plasma glucose, glycated hemoglobin, and triglycerides (Table 1b). This interaction consisted of the diabetes remission group, presenting with larger decreases in these variables, whereas smaller decreases or even no change (in the case of triglycerides) were observed in non-diabetic patients and in those in whom diabetes persisted after surgery.

### 3.2. Analysis of Plasma Proteins Changes in Response to Weight Loss Resulting from Bariatric Surgery

We separated the plasma proteins of all subjects obtained before and after surgery using 2D-DIGE gels. We visualized an average of 1540 spots per gel corresponding to the different plasma proteins. We detected 34 spots that reached statistically significant differences in the protein abundance using the standardized logarithm of abundance and the ratio of 1.4 or above that excluded our in-house experimental variation [24]. The results of this statistical analysis are described in detail in Supplementary Table S2 including the differences in protein abundance observed in all subjects before and after surgery, those observed between the groups of patients, and the interactions between diabetes and bariatric surgery (meaning different responses to weight loss among the groups of obese subjects).

**Figure 1:**
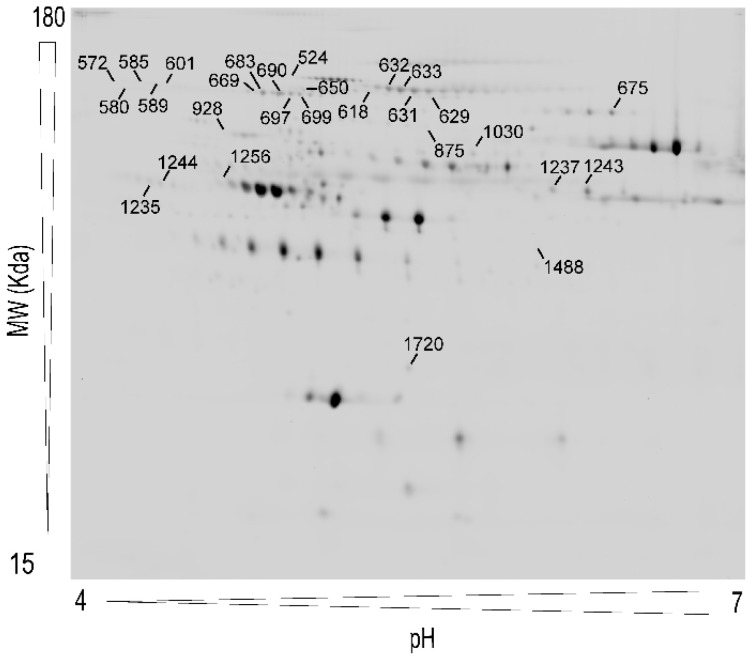
Representative two-dimensional differential in-gel electrophoresis (2D-DIGE) map of plasma protein extracts including the pick locations of the proteins identified in the study.

Figure 1 shows a representative 2D-DIGE map of plasma proteins including the point locations of these spots. MALDI-TOF/TOF analysis identified the proteins present in 28 of the 34 spots. Because different isoforms of the same protein may be found in more than one spot, these 28 spots corresponded to 13 proteins. Detailed information on the identification process of the 28 spots is summarized in Supplementary Table S1, including accession numbers, theoretical molecular weight, isoelectric point values, and parameters of the identification method.

**Figure 2:**
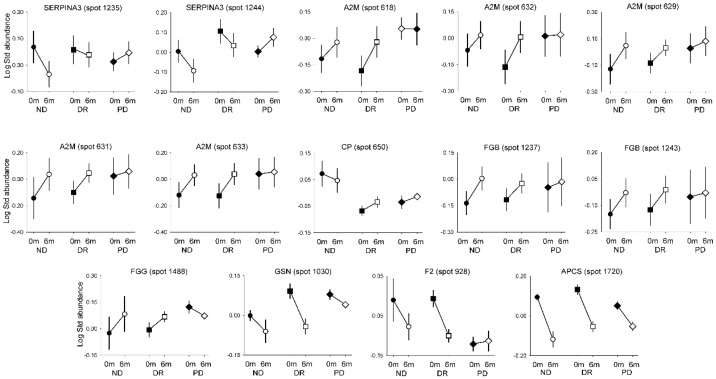
Interactions between changes in protein abundance before and after bariatric surgery, and groups classified by diabetes and its remission or persistence. ND, non-diabetic patients; DR, diabetes remission; PD, persistent diabetes. Data are means ± SEM.

The most relevant results for our aim were those resulting from the analysis of the interactions between the group of patients and the time of sampling, meaning the effect of surgically induced weight loss on protein abundance differed depending on the glucose tolerance of the obese subjects (Figure 2). We observed interactions in 14 spots from eight proteins: SERPINA3, prothrombin (F2), alpha-2-macroglobulin (A2M), fibrinogen beta chain (FBG), fibrinogen gamma chain (FGG), CP, gelsolin (GSN), and APCS. As shown in Figure 2, the abundance of SERPINA3 (spots 1235 and 1244), F2 (spot 928), and GSN (spot 1030) decreased after surgery in the non-diabetic (all spots) and diabetes remission groups (except for spot 1235), whereas no changes were observed in the persistent diabetes group. The abundance of A2M (spots 618, 631, 632, and 633), FGB (spots 1237 and 1243), and FGG (spot 1488) increased in the non-diabetic and diabetes remission groups after surgery; such an increase was not observed in patients with persistent diabetes. Moreover, the abundance of CP (spot 650) only increased in the diabetes remission group. Finally, the abundance of APCS (spot 1720) decreased after surgery in all groups, even though the magnitude of the decrease was larger in the non-diabetic and diabetes remission groups compared with the reduction observed in the persistent diabetes group.

When considering the effect of surgically induced weight loss in all subjects considered as a whole, regardless of their glucose tolerance, the comparison of the protein abundance before and 6 months after surgery revealed changes in six spots pertaining to three proteins: kininogen-1 (KNG1) and inter-alpha-trypsin inhibitor heavy chain H2 (ITIH2), increased after weight loss, whereas APCS decreased 6 months after surgery (Supplementary Figure S1 and Supplementary Table S2).

**Figure 3:**
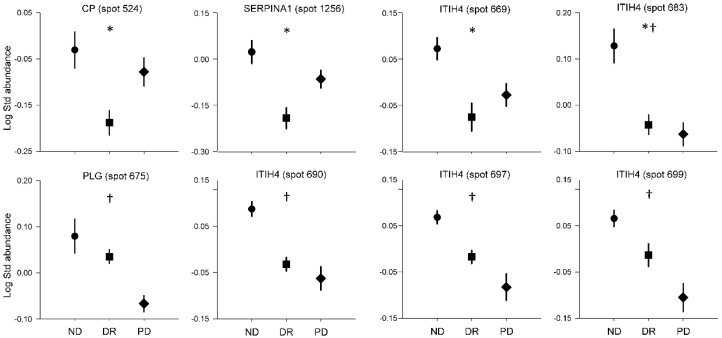
Differences between groups considering baseline and post-surgical values as a whole. ND, non-diabetic patients; DR, diabetes remission; PD, persistent diabetes. Data are means ± SEM. * *p* < 0.05, differences between ND and DR; ^†^ *p* < 0.05, differences between ND and PD.

Regarding differences among the non-diabetic, diabetes remission, and persistent diabetes groups, considering the samples obtained before and after surgery as a whole, we found different abundances in nine spots from five proteins: CP, SERPINA1, ITIH4, plasminogen (PLG), and FGG. As shown in Figure 3 and Supplementary Table S2, the protein abundances of CP (spot 524), SERPINA1 (spot 1256), and ITIH4 (spots 669 and 683) were reduced in the diabetes remission group compared with the non-diabetic group, while the persistent diabetes group showed intermediate values, except for spot 683. Moreover, the protein abundances of PLG (spot 675) and ITIH4 (spots 683, 690, 697, and 699) were lower in the persistent diabetes group compared with control patients, while the diabetes remission group showed intermediate values, except for spot 683. Finally, even though the overall GLM *p* value for FGG (spot 875) was 0.048, no statistically significant differences between the groups were found in the post hoc analyses.

We then tried to reproduce the changes in six of these proteins using immunoassays. The results mostly confirmed the interaction in CP abundance by showing a decrease after surgery in the diabetes remission group and an increase in the persistent diabetes group (Supplementary Figure S1). The concentrations of SERPINA1 confirmed the proteomic pattern of changes, although such differences between groups did not reach statistical significance. In contrast to the 2D-DIGE results, the plasma concentration of APCS increased six months after surgery, whereas no statistically significant differences were found between the groups or the time of sampling in SERPINA3, ITIH4, and KNG1 plasma levels (these molecules showed a considerably large individual variation when determined by immunoassays).

## 4. Discussion

Our present exploratory non-targeted study identified differences in the plasma proteomic profile of diabetic patients submitted to bariatric surgery in whom diabetes persisted or remitted after weight loss, using non-diabetic but similarly obese persons as controls.

The differences in the response of these subjects to surgically induced weight loss pertained to changes in the plasma abundance of SERPINA3, A2M, CP, FBG, FGG, GSN, F2, and APCS, proteins that are mostly related to pathways involved in acute-phase response, fibrinolysis, platelet degranulation, and blood coagulation. The pattern of the changes for most of these proteins was similar in non-diabetic patients and patients in whom diabetes remitted with weight loss, changes that were not observed, or were less pronounced, in patients with persistent diabetes. Even though our experimental design did not permit unraveling whether the observed changes in protein abundance represented any causative role or were merely associations, these proteins may be studied as potential biomarkers of diabetes remission after bariatric surgery.

These changes may suggest the involvement of the coagulation-fibrinolytic system, in conceptual agreement with the increased risk of atherothrombotic disease associated with type 2 diabetes [25]. Moreover, adipose tissue may play a significant role in stimulating hemostatic mechanisms by the production and secretion of factors influencing coagulation and fibrinolysis [26]. Serpins are a superfamily of proteins characterized by their common function as serine protease inhibitors, including those implicated in the coagulation process. SERPINA3 decreased after bariatric surgery only in the control and diabetes remission groups, suggesting an improvement in their inflammatory status [27,28] that apparently did not occur in the subjects with persistent diabetes. In line with this, other authors reported strong correlations between circulating SERPINA3 concentrations, glycated hemoglobin, and fasting insulin [29]. CP, a metalloprotein that binds most of the copper in plasma and is involved in the peroxidation of Fe(II) transferrin to Fe(III) transferrin, might be associated with the physiological adaptation of patients with T2D to the changes in iron metabolism after surgery. Although immunoassays did not confirm the interactions between group of subjects and surgery in terms of plasma abundance of CP and SERPINA3 levels, it is possible that the 2D-DIGE findings resulted from changes in certain isoforms of these proteins that might not be detectable by the immunoassays used here.

Gelsolin (GSN) is one of the most abundant actin-binding proteins involved in cell motility, shape, and metabolism, and also acts as a regulator of cell metabolism with substantial roles in many cellular pathways and interactions [30]. Plasma GSN levels were associated with several disorders such as amyloidosis, inflammation, and oncogenic transformation [31]. Khatri et al. [32] reported decreased circulating GSN levels in T2D patients compared with controls and that GSN replacement had beneficial effects in glucose tolerance in mouse models, similar to those observed with the drug sitagliptin. Joo et al. [33] found increased GSN in the white adipose tissue of obese rats fed a high-fat diet, resulting in greater body weight gain. We speculated that GSN may increase in obesity as a compensatory mechanism directed toward the maintenance of glucose tolerance, and that such a mechanism may be lost in the subset of obese subjects who develop diabetes. Normal glucose tolerance may require less compensation by GSN, explaining its decrease after bariatric surgery in our non-diabetic and diabetes remission subgroups. As an alternative explanation, identification by mass spectrometry may not distinguish between the alternative splicing derived cytoplasmic (89 kDa) and extracellular (83 kDa) isoforms [34]. It is possible that cytoplasmic GSN released from tissue cells interfered with measurements of plasma GSN levels.

After surgery, five spots of A2M of the non-diabetic group and the diabetes remission group increased, whereas only minor changes occurred in the persistent diabetes group. Alpha-2-macroglobulin inhibits a broad spectrum of proteinases as a protection against uncontrolled proteolytic activity [35]. This protein also functions as a carrier of specific growth factors and/or binding sites for receptors. Activation of A2M by proteases alters the interaction of A2M with these ligands and induces cell signaling [36]. A2M impacts in the transport and biological activity of insulin-like growth factors [37,38] and increased A2M serum levels, which characterize patients with diabetes [39,40].

Abnormalities in coagulation and fibrinolysis influenced the association of diabetes with prothrombotic risk [25]. Our results indicated that after bariatric surgery the abundance in two isoforms of FGG and one isoform of FGB increased in the control and remission groups, but not in the patients with persistent diabetes. Because post-translational modifications of fibrinogen affect clot formation, clot characteristics, and susceptibility to fibrinolysis [41], we speculate that patients with persistent diabetes display alternative post-translational modification of fibrinogens, which may account for the observed differences.

A significant decrease in abundance of CP, SERPINA1, ITIH4, PLG, and FGG was found in T2D patients compared with the non-diabetic group regardless of their changes before and after surgery. The co-regulation of these proteins may suggest the involvement of certain pathways in the association of diabetes with severe obesity. The primary target of SERPINA1 is elastase, but it also has a moderate affinity for plasmin and thrombin [42]. An imbalance between SERPINA1 and neutrophil elastase might contribute to the development of obesity and related inflammation, insulin resistance, and liver steatosis [43]. ITIH4 is a liver serine protease inhibitor that is highly expressed during liver development, and is an anti-inflammatory protein proposed to serve as a potential biomarker for acute ischemic stroke and rheumatoid arthritis [44,45]. Our results showed that four isoforms of ITIH4 and one isoform of SERPINA1 (spots 669 and 683) had lower abundance in the diabetes remission group than in the non-diabetic group and an intermediate value in the persistent diabetes group. We were not able to confirm the proteomic results by immunoassays because this method does not discriminate between the different protein isoforms or to the large inter-individual variability of plasma proteins previously described [46].

Regardless of diabetes and its remission, we found three proteins (KNG1, ITIH2, and APCS) whose abundance changed after 6 months of bariatric surgery when considering all patients as a whole. Kininogen interacts with plasma kallikrein to produce bradykinin and is involved in the contact activation of the blood coagulation process. Weight loss following very low calorie diets increased plasma KNG1 [46], in accordance with the increase observed in our patients following obesity surgery. ITIH2 function in metabolic diseases is unclear, although it might play some role as biomarker of diabetic retinopathy [47,48]. APCS levels decreased alter bariatric surgery, particularly in the non-diabetic and remission groups. APCS is involved in inflammatory processes and is specifically expressed and accumulated in atherosclerotic lesions [49]. Consistent with the decrease observed in our study after weight loss, a very low calorie diet also decreased plasma APCS [46]. The decrease in APCS after surgery in all three groups suggests an adaptation of the organism to the low-grade chronic inflammatory that underlies severe obesity. Taken together, these results may indicate that metabolic surgery induces alterations in these subjects regardless of abnormalities in glucose tolerance, probably as a result of marked weight loss. The effect of weight loss in plasma proteome was previously described after a very low diet and after bariatric surgery [46,50].

Our current study is not free of limitations. First, being a non-targeted exploratory study, we decided to study a small but rather homogeneous group of subjects submitted to bariatric procedures in whom only glucose tolerance varied, but not preoperative grade of obesity or amount of weight lost after surgery. We must highlight that our present results need confirmation in much larger series of patients to ascertain their possible role as biomarkers of diabetes remission after metabolic surgery. Additionally, the use of 2D-DIGE techniques imposes certain limitations on the precise identification of very hydrophobic proteins, proteins with very low concentration or molecular weight, and/or proteins showing extreme isoelectric point. Therefore, other complementary proteomic approaches may provide further insight into the molecular mechanisms of diabetes remission. Nevertheless, the use of proteomic approaches in our study allowed an accurate quantification of changes in protein abundance that might result from post-translational modifications, alternative transcription start sites, or alternative splice variants, and complement recent studies assessing the impact of metabolic surgery on adipose tissue proteome [51]. Our study also benefited from the inclusion of non-diabetic controls and from the use of plasma samples that are easy-to obtain and, therefore, are suitable for the development of biomarkers.

In conclusion, we identified several proteins that might be applicable in the biomarker discovery for diabetes remission after bariatric surgery, and may serve as the basis for future studies addressing the mechanisms underlying the normalization of glucose tolerance.

## Figures and Tables

**Table 1:** Clinical and biochemical characteristics of subjects before and 6 months after bariatric surgery.

	Non-Diabetic Patients		Diabetes Remission		Persistent Diabetes		Group	Surgery	Interaction
	Before Surgery (*n* = 6)	6 Months (*n* = 6)	Before Surgery (*n* = 7)	6 Months (*n* = 7)	Before Surgery (*n* = 4)	6 Months (*n* = 4)	*p* Value	*p* Value	*p* Value
Age	43 ± 10	NA	46 ± 8	NA	51 ± 5	NA	0.359	NA	NA
BMI (kg/m^2^)	43.4 ± 4.3	30.1 ± 4.6	46.5 ± 9.3	32.8 ± 5.9	44.1 ± 2.2	32.8 ± 4.8	0.663	<0.001	0.578
Decrease in BMI (kg/m^2^)	NA	13.3 ± 3.7	NA	13.7 ±3.9	NA	11.3 ± 3.8	0.578	NA	NA
Excess weight loss (%)	NA	74 ± 21	NA	67 ± 10	NA	68 ± 19	0.691	NA	NA
Waist (cm)	127 ± 13	101 ± 4	128 ± 17	110 ± 13	127 ± 10	110 ± 15	0.694	<0.001	0.439
Body fat (%)	50 ± 4	35 ± 7	48 ± 4	37 ± 3	53 ± 4	42 ± 9	0.363	<0.001	0.617
Glucose (mmol/L)	5.0 ± 0.6	4.3 ± 0.3	9.4 ± 3.7	4.7 ± 0.5	8.0 ± 1.2	8.3 ± 2.5	0.005 *^,†^	0.023	0.015
Insulin (pmol/L)	93 ± 27	30 ± 4	164 ± 89	35 ± 13	368 ± 589	64 ± 73	0.523	<0.001	0.400
HbA1c	5.6 ± 0.2	5.1 ± 0.2	7.7 ± 1.4	5.6 ± 0.3	7.6 ± 0.8	7.0 ± 1.3	0.001 *^,†^	<0.001	0.023
HOMA-IR	3.0 ± 1.0	0.8 ± 0.1	9.3 ± 0.4	1.1 ± 0.5	17.2 ± 26.3	3.1 ± 3.0	0.049 ^†^	<0.001	0.058
Triglycerides (mmol/L)	1.0 ± 0.3	1.0 ± 0.3	3.1 ± 1.9	1.1 ± 0.6	1.7 ± 0.8	1.6 ± 1.0	0.079	0.007	0.003
Total cholesterol (mmol/L)	5.0 ± 0.8	4.4 ± 1.1	5.4 ± 1.5	4.1 ± 1.0	4.4 ± 0.4	3.7 ± 0.9	0.444	0.004	0.645
HDL (mmol/L)	1.4 ± 0.2	1.6 ± 0.4	1.0 ± 0.3	1.2 ± 0.4	1.4 ± 0.2	1.3 ± 0.2	0.030 *	0.750	0.273
LDL (mmol/L)	3.2 ± 0.7	2.2 ± 1.0	3.3 ± 0.9	2.4 ± 0.9	2.3 ± 0.5	1.7 ± 0.5	0.115	0.022	0.744

Abbreviations: BMI, body mass index; HbA1c, glycated hemoglobin; HDL, high-density lipoprotein cholesterol; HOMA-IR, homeostasis model assessment of insulin resistance; LDL, low-density lipoprotein cholesterol; NA, not applicable. Data are means ± SD. The effects of group of subjects, before and after surgery, and the interaction of both effects were analyzed by repeated-measures GLM after applying logarithmic transformation as needed to ensure normality. * *p* < 0.05, differences between non-diabetic group and diabetes remission group; ^†^ *p* < 0.05, differences between non-diabetic group and persistent diabetes group.

## Data Availability

All data sets generated during and/or analyzed during the current study are not publicly available but are available from the corresponding author upon reasonable request.

## Supplementary Materials

The following are available online at https://www.mdpi.com/article/10.3390/jcm10173879/s1, explanation of experimental details, Figure S1: Measurement of plasma proteins by ELISA and immune nephelometry of several proteins identified by 2D-DIGE, Table S1: Detailed information of MS identification of proteins showing differences in plasma samples of non-diabetic patients (ND), diabetes remission group (DR), and persistent diabetes group (PD) submitted to bariatric surgery, as detected by 2D-DIGE, Table S2: Differences in protein abundance in plasma samples of non-diabetic patients (ND), diabetes remission group (DR), and persistent diabetes group (PD) submitted to bariatric surgery, as detected by 2D-DIGE, MIAPE document, and STROBE checklist.
