# Supplementary material for: Remission of Diabetes Following Bariatric Surgery: Plasma Proteomic Profiles
Source: J Clin Med. 2021 Aug 28;10(17):3879. doi: 10.3390/jcm10173879 (PMC8432028; doi:10.3390/jcm10173879)
Supplement: Supplementary file 1 [file jcm-10-03879-s001.zip › jcm-1312372-supplementary.pdf]

Supporting information

Methods

Sample preparation and two-dimensional differential in gel electrophoresis 2D-DIGE

For 2D-DIGE experiments, DeCyder 2D software v. 7.0 (GE Healthcare, Upsala, Sweden) differential in gel analysis (DIA) module was used for intra-gel co-detection of samples and internal standard protein spots allowing the detection of an average of 1540 spots with an 11% coefficient of variation (CV). Artefactual spots were filtered and removed.

Protein abundance in plasma before and after surgery was evaluated in three groups ofseverely obese patients: six metabolically healthy patients, seven patients with diabetes that remitted after surgery, and four patients with diabetes that did not enter remission after surgery. Accordingly, 34 samples were analyzed in 17 gels. Half of the samples from each of the groups were labeled with Cy3 dye and the other half with Cy5 dye to avoid any possible bias derived from labeling efficiency. All samples were pooled in equal protein amounts and labeled with Cy2 dye to serve as an internal standard for normalization in quantitative comparisons.

The 51 images derived from the 17 dyed gels were submitted to DeCyder's biological variation analysis (BVA) module for inter-gel matching of internal standard and samples across all gels. BVA performed comparative cross-gel statistical analyses of all spots based on spot volumes, permitting the detection of differences in protein abundance among experimental conditions.Differences are reported as volume ratios. We selected a 1.4-fold change as the cut-off that excluded an influence of experimental variability when comparing plasma samples in our laboratory using identical techniques.

DeCyder detected an average of 1540 spots per gel in a linear range (pH 4–7) with a molecular mass of 10-180 kDa. An average of 980 spots was matched on the gels (8.7% CV). Only spots present in at least 41 of the 51 gel images were considered for further analysis. Spots volumes of matched

protein spots were normalized and quantified. Matched spots and data quality were verified manually to exclude artifacts thereby avoiding false positive results. Normalized volume data for each spot were exported using DeCyder's XML toolbox. Only spots showing differences in protein abundance that were ≥1.4-fold and statistically significant *(P< 0.05)* were selected for identification by mass spectrometry (MS). For protein identification, MALDI-TOF MS analyses were performed in a 4800 Proteomics Analyzer MALDI-TOF/TOF mass spectrometer (Applied Biosystems, MDS Sciex, Toronto, Canada) at the Genomics and Proteomics Center, Universidad Complutense, Madrid.

ELISA and immunenephelometry assays

Six proteins identified in the non-targeted proteomic analysis were further assayed by appropriate immunoassays. We measured serum amyloid P (APCS), alpha-1-antichymotrypsin (SERPINA3), kininogen (KNG) and inter alpha globulin inhibitor H4 (ITIH4) concentrations using commercial ELISA kits ab137970-Serum amyloid P (APCS) human ELISA kit; ab171574-Alpha1-antichymotrypsin (SERPINA3) Human SimpleStep ELISA Kit,ab108875-kininogen (KNG) Human ELISA KIT Abcam plc, Cambridge, UK; SEH776Hu ELISA Kit for Inter Alpha Globulin Inhibitor H4 (ITIH4), Cloud-Clone Corp. Houston, USA]. Samples were assayed in duplicate. The lower limit of detection was 0.15 ng/ml for APCS, 88 pg/ml for SERPINA3, 0.01µl/ml for KNG and 0.17ng/ml for ITIH4. The mean intra- assay CVs were 4.9%, 3.1 %, 4.9% and 10% the mean inter-assay CVs were 7.1% ,4.4%, 7.1% and 12% for APCS, SERPINA3, KNG and ITIH4, respectively.

Circulating concentrations of ceruloplasmin (CP) and alpha-1-antitrypsin (SERPINA1) were assayed by a commercial immunonephelometry method (Dade Behring, Marburg, Germany) calibrated against the international reference material with intra-assay and inter-assay CVs of 1.8 % and 2.3 % for CP and 2.0 % and 3.2% for SERPINA1.

Supplementary Figure S1. Measurement of plasma proteins by ELISA and immunenephelometry of several proteins identified by 2D-DIGE.

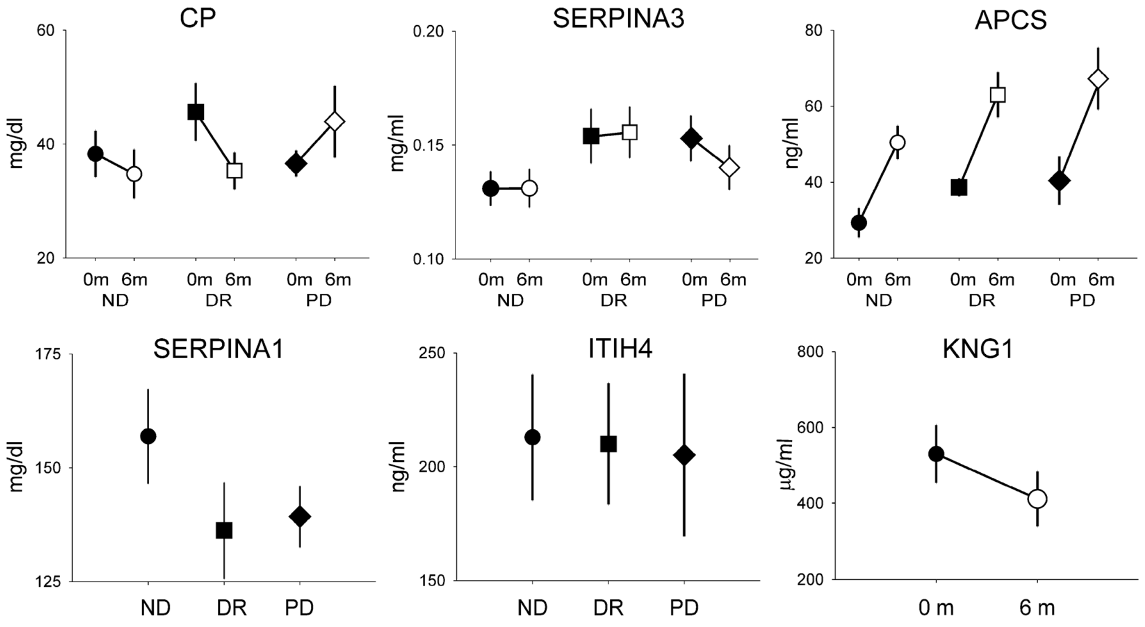

Supplementary Table S1 : Detailed information of MS identification of proteins showing differences in plasma samples of non-diabetic patients (ND), diabetes remissiongroup (DR) and persistent diabetes group (PD) submitted to bariatric surgery, as detected by 2D-DIGE.

				Identification Method						
				PMF			PMF and MS/MS			
**Pos**	**Protein ID**	**MW/pI**	**Protein Name**	**NP**	**Cov**	**Score**	**NP**	**FP**	**Score**	**Expect**
524	P00450	122/5.4	Ceruloplasmin	19	22	111				$1.60x10^{-07}$
572	P19823	106/6.4	Inter-alpha-trypsin inhibitor heavy chain H2				17	2	111	$1.60x10^{-07}$
580	P01042	63/6.3	Kininogen-1	20	35	98				$9.10x10^{-05}$
585	P01042	63/6.3	Kininogen-1	12	21	69				$2.40X10^{-03}$
589	P01042	63/6.3	Kininogen-1				25	3	227	$4.00X10^{-19}$
601	P01042	63/6.3	Kininogen-1				18	3	96	$4.50X10^{-06}$
618	P01023	164/6.0	Alpha-2-macroglobulin	23	21	111				$1.60X10^{-06}$
629	P01023	164/6.0	Alpha-2-macroglobulin	28	24	162				$1.30X10^{-30}$
631	P01023	164/6.0	Alpha-2-macroglobulin	43	36	341				$1.30X10^{-12}$
632	P01023	164/6.0	Alpha-2-macroglobulin	30	26	173				$1.00X10^{-13}$
633	P01023	164/6.0	Alpha-2-macroglobulin	27	24	153				$1.00X10^{-11}$
650	Q14624	103/6.5	Ceruloplasmin	14	14	66				$5.10X10^{-03}$
669	Q14624	103/6.5	Inter-alpha-trypsin inhibitor heavy chain H4				14	1	73	$1.00X10^{-03}$
675	P00747	90/7.0	Plasminogen	19	28	125				$6.40X10^{-09}$
683	Q14624	103/6.5	Inter-alpha-trypsin inhibitor heavy chain H4	28	30	197				$4.00X10^{-16}$
690	Q14624	103/6.5	Inter-alpha-trypsin inhibitor heavy chain H4	22	24	143				$1.00X10^{-10}$
697	Q14624	122/5.4	Inter-alpha-trypsin inhibitor heavy chain H4	14	14	66				$5.00X10^{-03}$
699	Q14624	103/6.5	Inter-alpha-trypsin inhibitor heavy chain H4	24	25	156				$5.10X10^{-12}$
875	P02679	52/5.4	Fibrinogen gamma chain	7	20	69				$2.50X10^{-03}$
928	P00734	71/5.6	Prothrombin				12	3	68	$3.00X10^{-03}$
1030	P06396	86/6.0	Gelsolin	25	35	203				$1.00X10^{-16}$
1235	P01011	48/5.3	Alpha-1-antichymotrypsin	21	44	154				$8.10X10^{-12}$
1237	P02675	56/8.5	Fibrinogen beta chain	33	66	269				$2.60X10^{-23}$
1243	P02675	56/8.5	Fibrinogen beta chain	28	61	218				$3.20X10^{-18}$
1244	P01011	48/5.3	Alpha-1-antichymotrypsin	20	44	141				$1.60X10^{-10}$
1256	P01009	47/5.4	Alpha-1-antitrypsin	19	49	181				$1.60X10^{-14}$
1488	P02679	52/5.3	Fibrinogen gamma chain				10	1	55	$7.00X10^{-02}$
1720	P02743	25/6.1	Serum amyloid P-component				13	3	387	$4.00X10^{-30}$

Pos: Position numbers correspond to the position of the proteins in the gel image (Figure 1).
MW/pI: Theoretical molecular weight (MW) in kDa and theoretical isoelectric point (pI).
PMF: peptide mass fingerprinting.
NP: Number of peptide mass values matched from MASCOT PMF.
Cov: Amino acid sequence coverage for the identified proteins. Score: MASCOT MS protein score, obtained from MALDI-TOF/TOF spectra from the top hit and the second one. Larger differences indicate a better result.
FP: Number of fragmented peptide masses by MS/MS.
Expect: Quality of an individual match. It is the number of times in the search we could expect to get a match with this score or higher by chance.

Supplementary Table S2 : Differences in protein abundance in plasma samples of non-diabetic patients (ND), diabetes remission group (DR) and persistent diabetesgroup (PD) submitted to bariatric surgery, as detected by 2D-DIGE.

			**GLM**	**Interaction**	**Group Differences**						**Effect of Bariatric Surgery**	
					**ND/PD**		**DR/PD**		**ND/DR**		**Before**	**After 6 Months**
Pos	Gene	Protein name	*P* value	*P* value	Ratio	*P* value	Ratio	*P* value	Ratio	*P* value	Ratio	*P* value
572	ITIH2	Inter-alpha-trypsin inhibitor heavy chain H2	0.247	0.564	-1.3	0.183	-1.4	0.111	1.1	0.774	**−1.5**	**<0.001**
601	KNG1	Kininogen-1	0.439	0.848	-1.1	0.616	-1.3	0.217	1.2	0.472	**-1.4**	**<0.001**
589	KNG1	Kininogen-1	0.499	0.763	-1.2	0.306	-1.3	0.298	1.0	0.981	**-1.5**	**<0.001**
580	KNG1	Kininogen-1	0.346	0.913	-1.4	0.162	-1.2	0.272	-1.1	0.646	**-1.4**	**0.002**
585	KNG1	Kininogen-1	0.195	0.832	-1.4	0.129	-1.4	0. 112	-1.0	0.928	**-1.6**	**0.005**
1256	SERPINA1	Alpha-1-antitrypsin	**0.005**	0.756	1.3	0.178	-1.3	0.047	**1.6**	**0.002**	-1.2	0.012
524	CP	Ceruloplasmin	**0.027**	0.750	1.1	0.428	-1.3	0.079	**1.4**	**0.009**	-1.3	0.001
875	FGG	Fibrinogen gamma chain	**0.028**	0.431	1.3	0.02	1.3	0.014	-1.0	0.992	-1.0	0.284
690	ITIH4	Inter-alpha-trypsin inhibitor heavy chain H4	**0.001**	0.133	**1.4**	**0.001**	1.1	0.363	1.3	0.001	-1.1	0.022
699	ITIH4	Inter-alpha-trypsin inhibitor heavy chain H4	**0.008**	0.174	**1.5**	**0.002**	1.2	0.054	1.2	0.071	-1.1	0.026
683	ITIH4	Inter-alpha-trypsin inhibitor heavy chain H4	**0.016**	0.677	**1.6**	**0.018**	1.0	0.987	**1.5**	**0.010**	-1.1	0.029
697	ITIH4	Inter-alpha-trypsin inhibitor heavy chain H4	**0.001**	0.065	**1.4**	**<0.001**	1.2	0.045	1.2	0.008	-1.1	0.024
669	ITIH4	Inter-alpha-trypsin inhibitor heavy chain H4	**0.048**	0.172	1.3	0.133	-1.1	0.443	**1.4**	**0.016**	-1.0	0.254
675	PG	Plasminogen	**0.036**	0.608	**1.4**	**0.013**	1.3	0.050	1.1	0.335	1.1	0.079
1244	SERPINA3	Alpha-1-antichymotrypsin	0.330	**0.043**	-1.2	0.394	1.1	0.745	-1.4	0.149	1.1	0.198
1235	SERPINA3	Alpha-1-antichymotrypsin	0.888	**0.035**	-1.1	0.867	1.1	0.802	-1.	0,.635	1.1	0.139
632	A2M	Alpha-2-macroglobulin	0.799	**<0.001**	-1.1	0.792	-1.2	0.521	1.1	0.698	-1.2	<0.001
633	A2M	Alpha-2-macroglobulin	0.783	**<0.001**	-1.2	0.549	-1.2	0.531	-1.0	0.989	-1.3	<0.001
618	A2M	Alpha-2-macroglobulin	0.541	**0.001**	-1.3	0.418	-1.3	0.280	1.0	0.780	-1.3	<0.001
629	A2M	Alpha-2-macroglobulin	0.768	**0.006**	-1.2	0.505	-1.2	0.546	1.0	0.906	-1.3	<0.001
631	A2M	Alpha-2-macroglobulin	0.849	**0.004**	-1.2	0.591	-1.2	0.662	-1.0	0.684	-1.3	<0.001
650	CP	Ceruloplasmin	0.048	**0.027**	1.2	0.092	-1.1	0.553	1.3	0.017	-1.0	0.290
1243	FGB	Fibrinogen beta chain	0.950	**0.002**	-1.2	0.754	-1.1	0.852	-1.1	0.882	-1.2	<0.001
1237	FGB	Fibrinogen beta chain	0.942	**0.016**	-1.2	0.784	-1.2	0.745	1.0	0.972	-1.2	<0.001
1488	FGG	Fibrinogen gamma chain	0.628	**0.044**	-1.1	0.467	-1.2	0.446	1.1	0.981	-1.1	0.066
1030	GSN	Gelsolin	0.174	**0.036**	-1.2	0.081	-1.1	0.451	-1.2	0.169	1.2	<0.001
928	F2	Prothrombin	0.295	**0.019**	1.2	0.144	1.2	0.200	1.0	0.824	1.2	0.001
1720	APCS	Serumamyloid P-component	0.341	**0.030**	-1.0	0.791	1.1	0.326	-1.1	0.177	**1.5**	**<0.001**

P values were obtained from univariate repeated-measures general linear model, and average ratios were obtained from DeCyder software.
